# Postoperative pain treatment with transmuscular quadratus lumborum block and fascia iliaca compartment block in patients undergoing total hip arthroplasty: a randomized controlled trial

**DOI:** 10.1186/s12871-021-01413-7

**Published:** 2021-07-10

**Authors:** Qin Xia, Wenping Ding, Chao Lin, Jiayi Xia, Yahui Xu, Mengxing Jia

**Affiliations:** 1 Department of Anesthesiology, Affiliated Hospital of Xuzhou Medical University, No.99, Huaihai West Road, Quanshan District, Jiangsu Province, 221000 China; 2 Department of Anesthesiology, Xuzhou Central Hospital, 199 Jiefang South Road, Quanshan District, Jiangsu Province, 221000 China; 3 Department of Anesthesiology, Xinhua Hospital, Shanghai Jiaotong University, 1665 Kongjiang Road, Yangpu District, Shanghai, 200082 China

**Keywords:** Multimodal analgesia, Transmuscular quadratus lumborum block, Fascia iliaca compartment block, Total hip arthroplasty

## Abstract

**Background:**

Patients often suffer moderate or even severe pain after total hip arthroplasty; such pain seriously affects early postoperative recovery. This study aimed to investigate the analgesic efficacy of ultrasound-guided transmuscular quadratus lumborum block combined with fascia iliaca compartment block for elderly patients undergoing total hip arthroplasty.

**Methods:**

Fifty-four patients scheduled for total hip arthroplasty were included in this randomized controlled study. The patients were randomly assigned to receive only transmuscular quadratus lumborum block (group Q) or transmuscular quadratus lumborum block combined with fascia iliaca compartment block (group QF) with ultrasound guidance. Postoperatively in both groups, paracetamol 1 g was regularly administered at 6 h intervals and patient-controlled intravenous analgesia was administered. The primary outcome was cumulative sufentanil consumption via patient-controlled intravenous analgesia 24 h postoperatively. The secondary outcomes included pain degree, time to the first analgesic requirement, joint range of motion, quality of recovery, and the incidence of postoperative complications.

**Results:**

Fifty patients were included, and their data were analyzed. The cumulative sufentanil consumption in group QF was significantly lower during the first 24 h after surgery than that in group Q, and the cumulative sufentanil consumption in group QF was reduced at 6–12 and 12–18 h after surgery. The postoperative pain intensity was lower in group QF than in group Q (linear mixed-effects model, the main effect of treatment: *P* < 0.001). Compared with group Q, group QF had higher quality of recovery and joint range of movement. The time to the first analgesic requirement was longer in group QF than in group Q (log-rank, *P* < 0.001). There was no statistically significant difference in complications postoperatively between the two groups.

**Conclusions:**

Our study provides a multimodal, opioid-sparing analgesic regimen for elderly patients undergoing total hip arthroplasty. The combination of transmuscular quadratus lumborum block and fascia iliaca compartment block provides a significant advantage for early postoperative functional recovery. Further studies are required to confirm the minimum effective dose.

**Trial registration:**

The study was registered on the 21st December 2020 (retrospectively registered) on the Chinese Clinical Trial Registry: ChiCTR2000038686.

## Background

With the Chinese population becoming an aging society, elderly patients are often troubled by joint degeneration, osteoarthritis, and fracture [1]. Generally, total hip arthroplasty (THA) is the common method to treat severe hip diseases and reconstruct joint function; however, the incidence and degree of postoperative pain are closely related to postoperative cardio-cerebrovascular complications and early postoperative recovery quality [2, 3]. A standardized, multimodal analgesic regimen is an essential and central element of ERAS pathways [4]. PROSPECT 2010 guidelines recommend various approaches, such as intravenous analgesia, epidural analgesia, local anesthetic infiltration techniques, and peripheral nerve block (PNB), that aim to minimize THA perioperative pain in elderly patients [5]. Nevertheless, there is no consensus on the optimal analgesic scheme for total hip arthroplasty. Postoperative pain management and minimization of opioid administration remain the primary perioperative challenges for elderly patients [6].

Opioids are the primary means of postoperative intravenous analgesia [7]. However, opioid-related adverse effects, such as postoperative nausea and vomiting (PONV), respiratory depression, and impaired gastrointestinal function, may weaken postoperative recovery quality [6]. Among many opioid-sparing regional anesthesia technologies for patients undergoing THA, time-tested epidural anesthesia contributes to pain relief [8]. Nevertheless, epidural anesthesia use has become limited in elderly patients due to lumbar degenerative disease and the wide application of preoperative anticoagulants [8]. Currently, PNB is an essential part of perioperative multimodal analgesia, providing site-specific, rapid-onset analgesia and attracting increasing attention [9].

Børglum [10] et al. first reported that the transmuscular quadratus lumborum block (T-QLB) was in 2013. Patients comparing T-QLB to lumbar plexus blocks for THA showed equivalent analgesia with similar opioid requirements and pain scores postoperatively in a retrospective cohort study [11]. Recently, a clinical study [12] showed that T-QLB could provide effective analgesia with opioid-sparing after THA. Similar results were demonstrated by Tulgar [13] et al. and Hockett [14] et al.

Fascial iliac compartment block (FICB) is an easier way to relieve patients’ THA-related pain than the anterior approach of the lumbar plexus, especially in emergency surgery [15]. Theoretically, in addition to the femoral and lateral femoral cutaneous nerves, FICB is capable of blocking the obturator nerve. Hebbardet [16] et al. reported a ‘longitudinal supra-inguinal approach’ (S-FICB) to improve the spread of local anesthetic (LA) and the success of FICB. This is mainly because the femoral cutaneous nerve has an inconsistent course, with variable branching below the inguinal ligament.

It is challenging to meet patients' requirements by performing single-shot PNB in THA, with the innervation involved in THA being complex [17]. Previous studies [11, 12, 15, 18] focused more on the application of single-shot PNB (such as lumbar plexus block, sacral plexus block, femoral nerve block, FICB, T-QLB) in total hip arthroplasty. These factors may increase the risk of local anesthetic overdose, high anesthetic concentration, nerve injury, and local anesthetic intoxication. The muscle and skin sensation involved in THA surgical incision is innervated by branches of superior cluneal nerves, the subcostal, iliohypogastric, ilioinguinal, femoral, obturator, sciatic, and lateral femoral cutaneous nerves [12]. A cadaver study [19] showed the spread of a dye around the subcostal nerve, iliohypogastric nerve, ilioinguinal nerve, genitofemoral nerve, and caudal spread to L2–L3 dermatomes by T-QLB. S-FICB can produce a more complete sensory block of the femoral, obturator, and lateral femoral cutaneous nerves [17, 18]. We hypothesized that the combined application of T-QLB and FICB could optimize the effect of nerve block in the aspects of block range and degree, further reduce or eliminate the pain caused by noxious stimulation, and achieve a better analgesic effect.

The aim of the study was to compare the impact of T-QLB and T-QLB + FICB on postoperative sufentanil consumption, pain scores, joint range of motion, quality of recovery, and the incidence of postoperative complications in patients undergoing THA.

## Methods

This study was approved by the Affiliated Hospital of Xuzhou Medical University's ethics committee. This manuscript adheres to the applicable CONSORT guidelines. This study was a single-center, prospective, single-blind, randomized controlled trial. Fifty-four elderly patients scheduled for elective total hip arthroplasty in the Affiliated Hospital of Xuzhou Medical University from November 2019 to August 2020 provided written informed consent.

### Study participants

The inclusion criteria were as follows: patients who (1) underwent primary unilateral THA; (2) aged 65–80 years old; and (3) American Society of Anesthesiologists' (ASA) physical status II-III. The exclusion criteria were as follows: (1) severe abnormal coagulation function; (2) puncture site infection; (3) morbid obesity (BMI > 35 kg/m2); (4) unable to cooperate with researchers for any reason; (5) allergy to local anesthetics; and (6) chronic pain, long-term use of analgesics or other psychotropic drugs. Patients who violated the scheduled postoperative analgesia program, were transferred to the ICU after surgery, and had a failed block were also excluded from the analysis.

### Randomization and blinding

An anesthesia assistant (not involved in the study) generated random numbers with a 1:1 ratio for group Q or group QF using a computerized random-number generator. The randomization sequence was put into sealed opaque envelopes and drawn up by an experienced anesthetist who performed the block and anesthesia induction. An investigator assessing the block quality was blinded to the group allocation. A resident anesthetist blinded to the randomization was responsible for the collection of intraoperative data. Another investigator who was independent of the group allocation collected postoperative data. Finally, a statistician masked the entire process and performed the statistical analysis.

### Study procedures

After entering the anesthesia preparation room, subjects were monitored with electrocardiography, invasive arterial blood pressure, and pulse oximetry. The blocks were performed on the side of surgery with a 22G/100-mm Stimuplex block needle (Braun, Ogaki, Japan) using an ultrasound machine (diagnostic ultrasound system, model Wisonic Navi s, Shenzhen Wisonic Medical Technology Co., Ltd., China). An experienced anesthesiologist performed all block procedures before anesthesia induction.

**Fig. 1:**
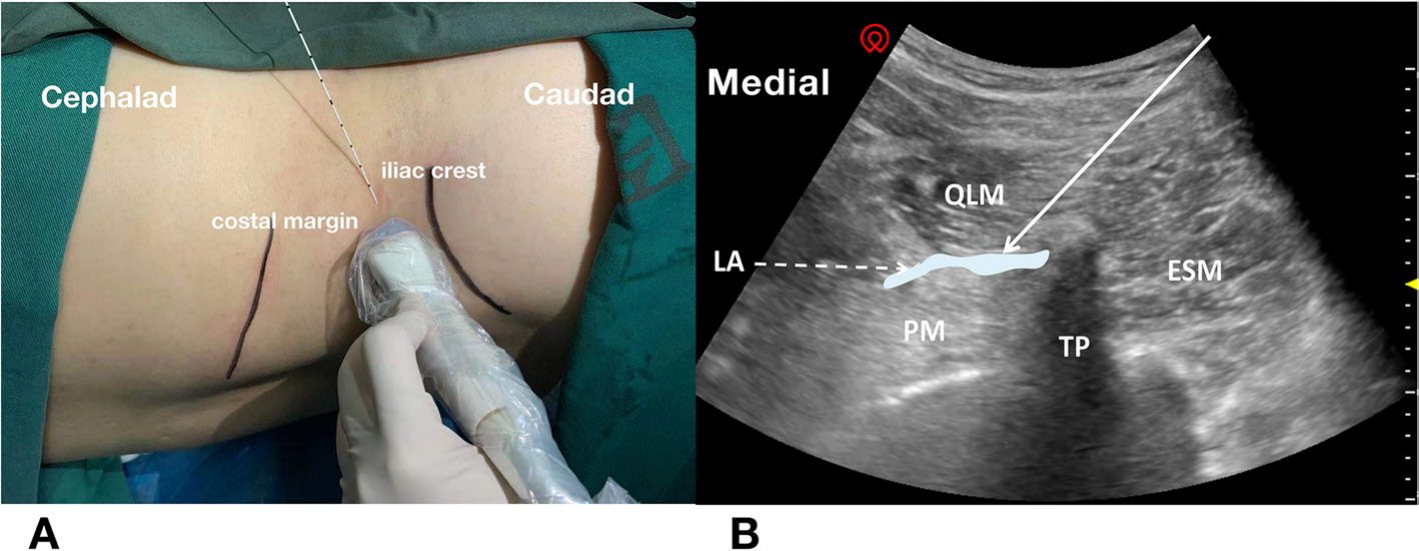
**A**: Posture and injection approach of transmuscular quadratus lumborum block; **B**: Ultrasound image of transmuscular quadratus lumborum block. Solid arrow indicates needle trajectory and injection point between QLM (quadratus lumborum muscle) and PM (psoas major muscles); dashed line indicates the spread of the LA (local anesthetic); blue:local anesthetic; ESM: erector spinae muscle; TP: transverse process

In group Q, T-QLB was performed in the lateral position, and the surgical side was nondependent, with lower extremity flexion [20]. The skin was sterilized twice with chlorhexidine. The low-frequency curvilinear ultrasonographic probe was placed transversely cranially to the iliac crest and at the posterior axillary line level and then moved to the dorsal side (Fig. 1). After the probe visualizes the ‘Shamrock sign,’ composed of the quadratus lumborum muscle (QLM), psoas major (PM), and erector spinal muscles [21]. Infiltrating the skin with 2 ml of 2% lidocaine. Using an in-plane approach, a 22G/100-mm Stimuplex block needle was inserted on the posterior corner of the convex probe. When the correct needle position was achieved via repeated negative aspiration tests and hydro-separation, a total of 40 mL of 0.375% ropivacaine was injected incrementally between the QLM and PM.

**Fig. 2:**
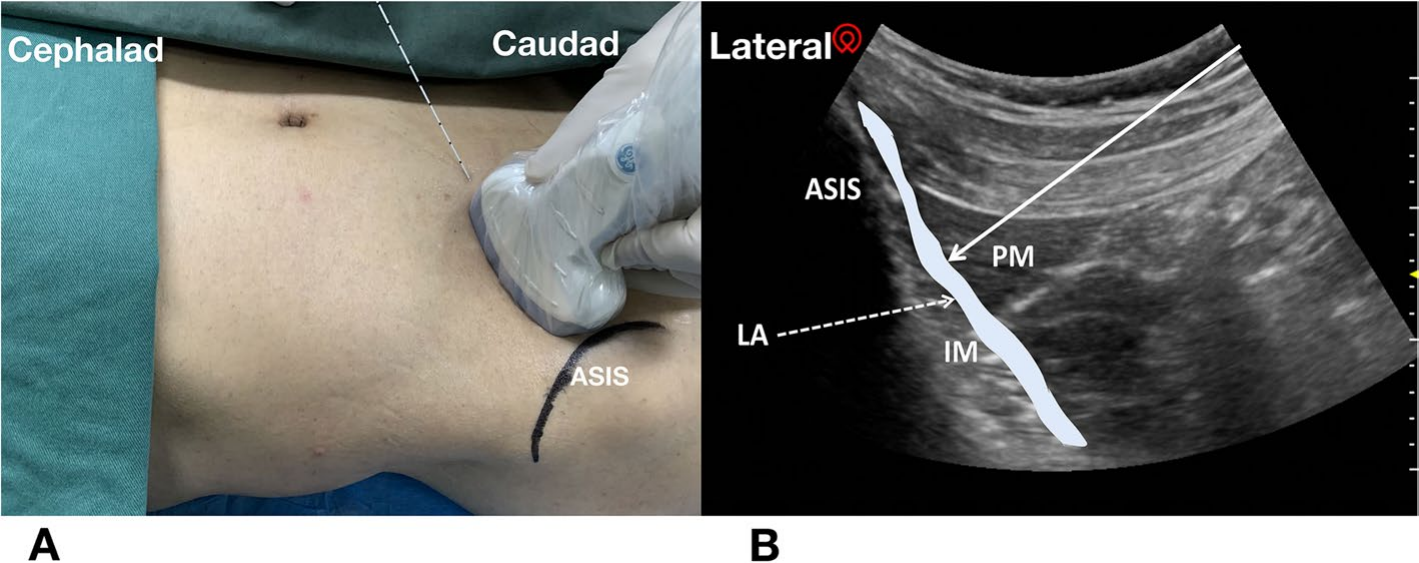
**A**: Posture and injection approach of fascia iliaca compartment block; **B**: Ultrasound image of fascia iliaca compartment block. Solid arrow indicates needle trajectory and injection point between fascia iliaca and iliac muscle (IM); dashed line indicates the spread of the LA (local anesthetic); blue:local anesthetic; ASIS, anterior superior iliac spine; PM, psoas major muscles

In group QF, FICB was administered in the supine position with the technique used by Hebbard and colleagues [16]. Initially, the low-frequency curvilinear ultrasonographic probe was placed at the inguinal ligament crease to identify the femoral artery and sartorius muscle by short-axis scanning and then move the probe cranially to the anterior superior iliac spine level. Rotating the probe 90 to 120° counterclockwise, the external oblique muscle, internal oblique muscle, transverse abdominal muscle aponeurosis, PM, and iliac fascia covering the iliac muscle were visualized; the latter was the final probe position (Fig. 2). After skin infiltration with 2 ml of 2% lidocaine, a 22G/100-mm Stimuplex block needle was advanced in an in-plane technique to the point that the fascia iliaca was penetrated and hydro-separation. Once tip position security was confirmed, 20 ml of 0.375% ropivacaine was injected incrementally into the surface of the iliacus muscle. After that, the patient switched to a lateral position, and QLB was performed. The specific procedure was the same as that in group Q, and 20 ml of 0.375% ropivacaine was injected.

Thirty minutes after performing the block, the block effect was evaluated by a masked investigator with pin-prick sensation in each dermatomal distribution of the obturator nerve, lateral femoral cutaneous nerve, and femoral nerve. Pain to pin-prick was graded according to a 3-point scale: 0 = pain disappearance (no sensation of pain), 1 = hypoesthesia (decreased sensation of pain compared to the opposite side), 2 = normal sensation [22]. If the three branches of the innervated area were less than or equal to 1 point, it was considered block effective. Patients with a score of 2 were considered block failure and then excluded from the study.

### Anesthesia

All subjects received standardized general anesthesia as follows: induction with midazolam 0.05 mg/kg, etomidate 0.3 mg/kg, sufentanil 0.5 μg/kg, and cis-atracurium 0.15 mg/kg; insertion of the laryngeal mask airway (LMA). Adjust respiratory parameters to maintain 35–40 mmHg of PetCO2 (partial pressure of end-tidal carbon dioxide). Then, anesthesia was maintained with propofol 3 mg/(kg·h) and remifentanil 0.3 μg/(kg·min), and the infusion rate of propofol was adjusted to keep the bispectral index (BIS) within 40–60. If the mean arterial pressure (MAP) increased by more than 20% compared with baseline, a 0.5 μg/kg supplemental dose of remifentanil was provided, and increasing the infusion rate of remifentanil by 0.05 μg/(kg·min), and nicardipine or esmolol was administered as appropriate. After completion of the surgery, patients were transferred to the postanesthesia care unit (PACU) and received intravenous tropisetron 4 mg and paracetamol 1 g. When the patient was fully awake and meets the extubation principle, remove the LMA.

### Postoperative pain management

Postoperative multimodal analgesia included oral nonsteroidal anti-inflammatory drugs, patient-controlled intravenous analgesia (PCIA), and rescue analgesia. The patient received oral paracetamol 1 g regularly at 6 h intervals. The PCIA pump was composed of sufentanil 100 μg + tropisetron 8 mg, diluted with normal saline to 100 ml, programmed to deliver 2 ml per dose with a lock-time of 15 min, without a background infusion. Pain was assessed using the numerical rating scale (NRS) from 0 to 10 (0 = no pain, 10 = most severe pain). The subjects were trained before the operation, and the PCIA pump was used when the patient reported NRS > 3. Nonetheless, if the pain could not be relieved by PCIA, tramadol 25 mg i.v. was prescribed as rescue analgesia.

### Outcome measurements

Outcome assessment was conducted by investigator members trained before the study and independent of the group allocation. The primary outcome was cumulative sufentanil consumption via PCIA in the first 24 h postoperatively. The secondary outcomes including (1)sufentanil consumption at 6 h intervals (0–6, 6–12, 12–18, and 18–24 h) after surgery (μg), (2)the pain scores both at rest (supine position) and during movement (defined as lifting 15° on the affected limb in supine position) were assessed with NRS at 2, 6, 12, 18, and 24 h postoperatively(NRS 0–10/10),(3)time to the first analgesic requirement (time from the completion of the block to the first PCIA opioid bolus) (min), (4) quality of recovery-15 (Qor-15 scale) scores [23] at 24 h and 48 h after surgery, (5) the maximal flexion and abduction range of movement (ROM) of the hip joint at 12, 24, 48 and 72 h postoperatively(°), (6) number of people requiring rescue analgesia, and (7) incidence of nausea and vomiting (yes/no).

### Statistical analysis

The sample size was calculated based on our preliminary study. Our preliminary experience with T-QLB showed that the cumulative sufentanil consumption was 46.4 ± 17.5 μg (mean ± standard deviation [SD]) in the first 24 h postoperatively. The cumulative sufentanil consumption was reduced by roughly one-third when patients were receiving T-QLB combined with FICB. Thus, we supposed that sufentanil consumption in the first 24 h would be reduced by a third in group QF in this study. The sample size calculated by PASS 15.0 software (NCSS, LLC, Kaysville, USA) was 24 individuals per group (with *α* = 0.05, power = 0.8). Considering the loss-to-follow-up rate of approximately 10%, we enrolled 54 subjects.

Data were analyzed using SPSS statistical software 25.0 (SPSS for Windows, ver. 25.0). The Kolmogorov–Smirnov test was used to evaluate the normal distribution of data. Continuous data are presented as the mean and SD or median and interquartile range. Standard hypothesis tests (2-sided t-test or Mann–Whitney U test) were performed to analyze baseline characteristics and outcome parameters. Categorical data are presented as n (%) and were analyzed by using chi-square tests or Fisher’s exact test. Kaplan–Meier curves and log-rank tests were performed to analyze the time-to-event data. The repeated measurement data (such as sufentanil consumption at 0–6, 6–12, 12–18, and 18–24 h after surgery, pain score during rest and movement at 2, 6, 12, 18, 24 h after surgery, and other repeated measurement data involved in this study) were compared using a linear mixed-effects model (LMM) [24]. The linear mixed-effects model was performed using the lmerTest package in R software (R version 3.6.1). The group, time (modeled as a categorical variable) and group-by-time interaction were fixed effects, and the random effect was a random intercept for subjects.

## Results

**Fig. 3:**
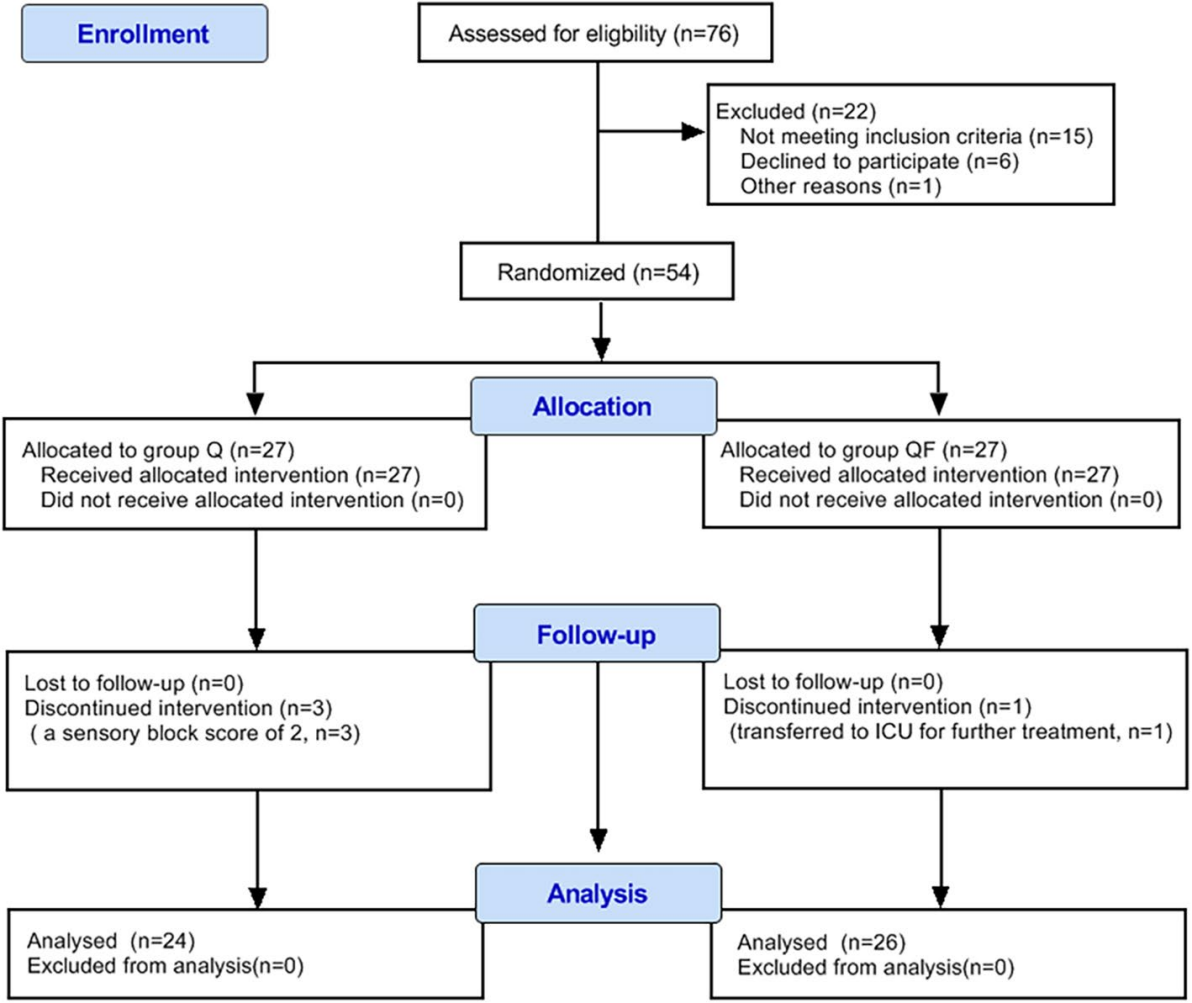
Consolidated Standards of Reporting Trials (CONSORT) flow diagram

Between November 2019 and August 2020, 76 subjects were screened for study participation. Of these, 54 subjects were included and randomly assigned to receive either T-QLB (n = 27) or T-QLB combined with FICB (n = 27). Among them, three subjects had a sensory block score of 2 after performing the block, and one subject subjects were transferred to the ICU for further treatment after surgery (Fig. 3). Eventually, fifty subjects completed the study and were analyzed as per-protocol (24 in group Q, 26 in group QF). The patient demographics and surgery time in the two groups were comparable (Table 1). There was no significant difference between the two groups regarding the incidence of PONV (*P* > 0.05) (Table 2). We did not notice any relevant complications, such as cardio-cerebrovascular complications, hypotension, or urinary retention, among the patients.

**Table 1:** Patient demographics and perioperative characteristics

	Group Q (n = 24)	Group QF (n = 26)
Age (y), mean ± SD	70.88 ± 3.70	69.88 ± 2.76
Sex		
Male, n(%)	10(41.7)	11(42.3)
Female, n(%)	14(58.3)	15(57.7)
BMI (kg/m^2^), mean ± SD	23.52 ± 2.73	22.31 ± 3.38
ASA		
II, n(%)	11(45.8)	12(46.2)
III, n(%)	13(54.2)	14(53.9)
Preoperative NRS at rest, (median, IQR)	3(3–4)	3(2.5–4)
Preoperative NRS at activity, (median, IQR)	6(5–7)	6(5–7)
Qor-15 score, mean ± SD	96.08 ± 1.58	95.19 ± 1.13
Hypertension, n(%)	9(37.5)	11(40.7)
Diabetes, n(%)	7(29.2)	9(34.6)
Duration of surgery (min), mean ± SD	105.50 ± 11.24	102.23 ± 10.52

*Abbreviations*: *BMI* body mass index, *ASA* American Society of Anesthesiologists, *NRS* numerical rating scale, *Qor-15 score* quality of recovery-15 score, *SD* standard deviation, *IQR* interquartile range

**Table 2:** Comparision of the subject primary outcome, and secondary outcomes

	Group Q (n = 24)	Group QF (n = 26)	*P*-value
Postoperative 24 h sufentanil dosage(μg), (mean ± SD)^a^	49.29 ± 16.76	31.42 ± 18.81	< 0.001^*^
Sufentanil dosage at 6 h interval(μg), (mean ± SD)^b^			
0–6 h	2.71 ± 2.33	0 ± 0	0.122
6–12 h	8.20 ± 5.07	4.68 ± 3.67	0.044^*^
12–18 h	18.89 ± 7.87	10.86 ± 7.65	< 0.001^*^
18–24 h	19.48 ± 7.17	16.15 ± 9.34	0.058
Remifentanil dosage(mg), (mean ± SD)^a^	1.62 ± 0.52	1.17 ± 0.50	0.003^*^
Propofol dosage(mg), (mean ± SD)^a^	337.08 ± 48.82	355.01 ± 52.78	0.230
Time to removal of laryngeal mask(mins), (mean ± SD)^a^	17.92 ± 5.98	10.58 ± 3.74	0.001^*^
Time to the first analgesic require(mins), (mean ± SD)^c^	680.33 ± 311.95	1147.73 ± 351.93	< 0.001^*^
Number of use remedial analgesia, n(%)^d^	7(29.2)	2(7.69)	0.063
The incidence of PONV, n(%)^d^	7(29.2)	4(14.8)	0.210

*Abbreviations*: *Qor-15 score* quality of recovery-15 score, *PONV* postoperative nausea and vomiting, *SD* standard deviation

^a^ Student’s *t*-test

^b^ Linear mixed-effects models

^c^ Log-rank test

^d^ χ^2^ tests or Fisher’s exact tests

^*^ There were significant differences between the two groups (*P* < 0.05)

### Opioid consumption

Compared with group Q, the intraoperative dosage of remifentanil and cumulative sufentanil consumption in group QF were significantly lower in the first 24 h after surgery (*P* < 0.001) (Table 2). The LMM was used to examine the changes in cumulative sufentanil consumption over the first 24 h after the operation. The time-by-group interaction was statistically significant (*P* = 0.022, LMM). There was a significant difference in sufentanil consumption between the two groups (*P* < 0.001, LMM), and the sufentanil consumption in group QF was less than that in group Q at 6–12 and 12–18 h postoperatively (*P* = 0.044 and *P* < 0.001, respectively, LMM). Moreover, the number of people requiring rescue analgesia in group QF was fewer than that in group Q (*P* < 0.001).

### Pain intensity

**Fig. 4:**
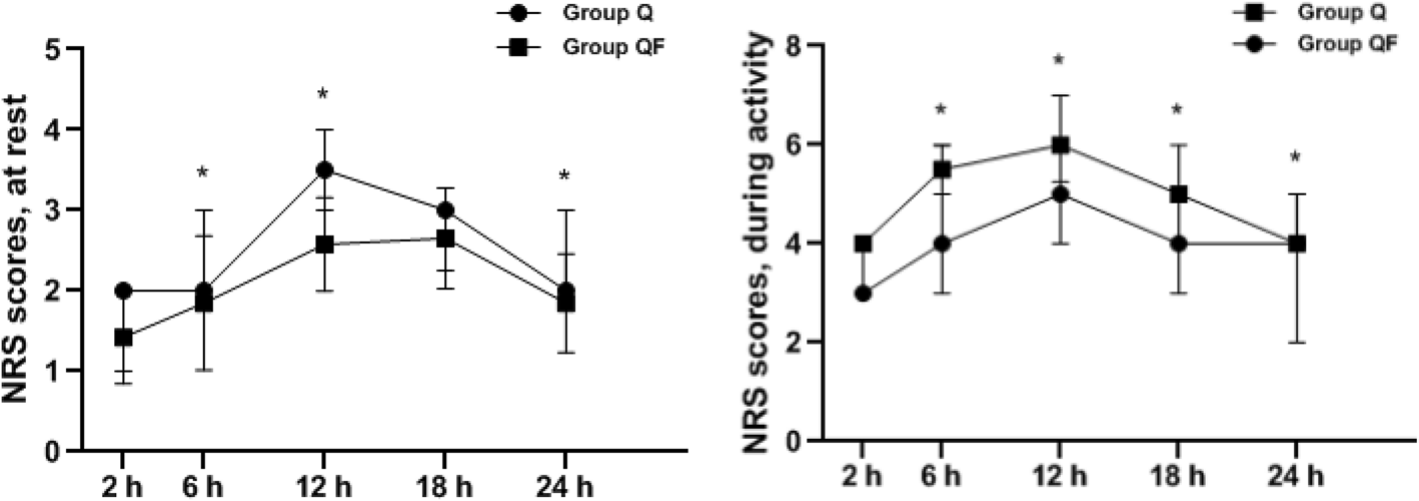
Numeric Rating Scores at rest (left) and during movement (right). NRS, numeric rating scores. Data are expressed as median and interquartile range. ^*^: *P* < 0.05

The pain scores at rest and movement for different time points are shown in Table 3. The change in the NRS scores over time in group QF was significantly different from the change in group Q both at rest and during movement (*P* < 0.001 and *P* < 0.001, respectively, LMM). Separate analyses per time point showed that, compared with group Q, the NRS scores at rest in group QF were significantly lower at 6, 12, and 24 h after surgery (*P* = 0.006, *P* < 0.001, and *P* < 0.021, respectively, LMM) (Fig. 4a), and the NRS scores during movement were significantly lower at 6, 12, 18, and 24 h after surgery in group QF than in group Q (*P* < 0.001, *P* < 0.001, *P* < 0.001, and *P* < 0.001, respectively, LMM) (Fig. 4b).

**Table 3:** Comparision of the pain intensity at rest and at activity

	Group Q (n = 24)	Group QF (n = 26)	*P*-value
NRS at rest, (median, IQR)^b^			
2 h	2(1–2)	1.5(1–2)	0.294
6 h	2(2–3)	2(1–3)	0.006^*^
12 h	3.5(3–4)	2.5(2–3)	< 0.001^*^
18 h	3(2–3)	2.5(2–3)	0.114
24 h	2(2–3)	2(1–2)	0.021^*^
NRS at activity, (median, IQR)^b^			
2 h	4(3–4)	3(3–4)	0.095
6 h	5.5(4–6)	4(3–5)	< 0.001^*^
12 h	6(5–7)	5(4–6)	< 0.001^*^
18 h	5(5–6)	4(3–5)	< 0.001^*^
24 h	4(4–5)	4(2–4)	< 0.001^*^

*Abbreviations*: *NRS* numerical rating scale, *IQR* interquartile range

^b^ Linear mixed-effects models

^*^ There were significant differences between the two groups (*P* < 0.05)

### Time to the first analgesic requirement

**Fig. 5:**
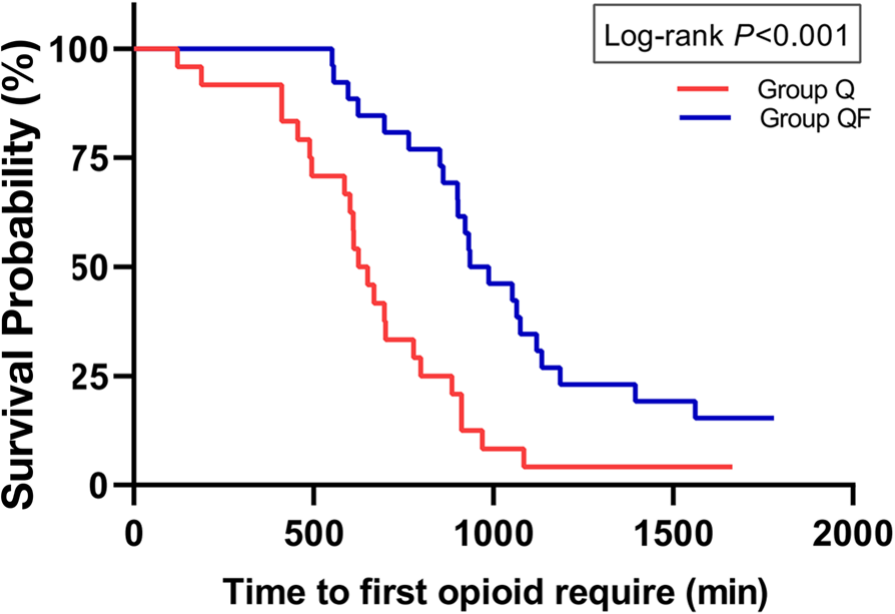
Kaplan–Meier curves for time to first opioid request

Kaplan–Meier survival curves of elapsed time showed that the time between completion of the block and the time to the first analgesic requirement was significantly longer in group QF than in group Q (*P* < 0.001) (Fig. 5). In addition, three of 26 patients distributed to group QF did not need additional opioid analgesia during the first 24 h after surgery.

### Range of motion

**Fig. 6:**
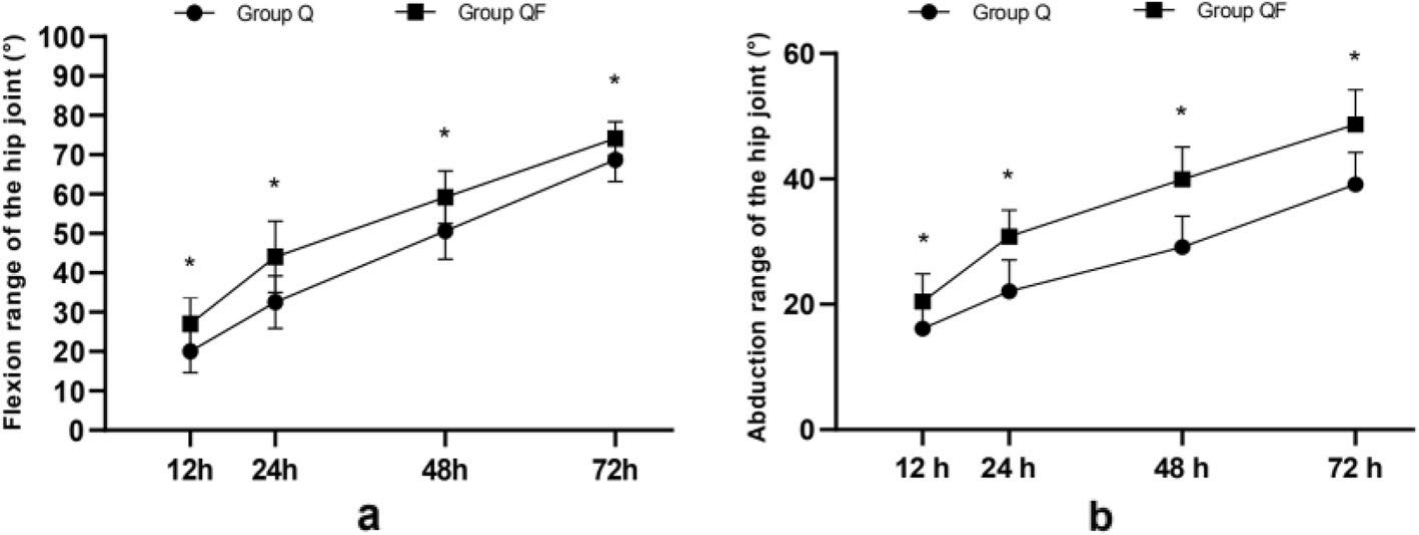
The maximum flexion (left) and abduction (right) ROM of the hip joint at 12 h, 24 h, 48 h and 72 h after surgery. ROM, range of movement. Data are expressed as mean ± SD. ^*^: *P* < 0.05

The maximal flexion (Fig. 6a) and abduction (Fig. 6b) ROM of the hip joint are shown in Table 4. The change over time in group QF was significantly different from the change in group Q both at extension and abduction (*P* < 0.001 and *P* < 0.001, respectively, LMM). Compared with group Q, the ROM was increased in group QF at the time of each clinical evaluation time.

**Table 4:** Comparison of hip ROM and Qor-15 score between the two groups

	Group Q(n = 24)	Group QF(n = 26)	*P*-value
hip flexion, ROM(°), (mean ± SD)^b^			
12 h	20.04 ± 5.42	27.08 ± 6.69	< 0.001^*^
24 h	32.54 ± 6.64	44.04 ± 9.12	< 0.001^*^
48 h	50.63 ± 7.20	59.19 ± 6.66	< 0.001^*^
72 h	68.71 ± 5.51	74.15 ± 4.32	0.004^*^
hip abduction, ROM(°), (mean ± SD)^b^			
12 h	16.13 ± 4.96	20.42 ± 4.46	0.002^*^
24 h	22.08 ± 4.99	30.85 ± 4.20	< 0.001^*^
48 h	29.13 ± 5.32	39.92 ± 5.15	< 0.001^*^
72 h	39.13 ± 5.11	48.65 ± 5.56	< 0.001^*^
Qor-15 score, (mean ± SD)^b^			
24 h after surgery	91.50 ± 5.71	100.04 ± 6.41	< 0.001^*^
48 h after surgery	101.71 ± 6.32	112.15 ± 5.88	< 0.001^*^

Abbreviations: *ROM* range of motion, *Qor-15 score* quality of recovery-15 score, *SD* standard deviation

^b^ Linear mixed-effects models

^*^ There were significant differences between the two groups (*P* < 0.05)

### Quality of recovery

The preoperative QoR-15 score in the two groups was not statistically significant. The increase in the Qor-15 score in group QF differed significantly from the change in group Q over the study period of 48 h (*P* < 0.001, LMM). The QoR-15 score of patients were significantly higher in group QF at 24 h and 48 h than in group Q (*P* < 0.001 and *P* < 0.001, respectively, LMM) (Table 4).

## Discussion

Our results showed that compared with single-shot T-QLB alone, the combination of T-QLB and FICB could reduce sufentanil consumption by 36% at 24 h postoperatively, significantly decrease the pain score, increase the early postoperative range of motion and improve the early quality of recovery without increasing complications.

Accumulating published data [13–15] were dedicated to exploring more effective multimodal analgesia with opioid-sparing. However, hip innervation is complex, with contributions from many nerve components [17]. Birnbaum [25] et al. reported that the nerves involved in THA incision pain mainly included the subcostal nerve, iliohypogastric nerve, ilioinguinal nerve, femoral nerve, lateral femoral cutaneous nerve, obturator nerve, and sciatic nerve. Additionally, the latest studies [15, 26] indicated that the femoral nerve, dominating the hip joint, branches at a higher position, and the location of the lateral femoral cutaneous nerve under the inguinal ligament have significant anatomical variability. It is difficult for single-shot PNB to meet the analgesic requirements of patients.

Previous studies [14–16] have shown that T-QLB and FICB can relieve postoperative pain after THA. T-QLB provides pain relief over the incision area for patients undergoing THA, mainly through blockade of the T10-L3 nerve territories and dermatomal tissue [20]. Kadam [27] et al. found that single-shot T-QLB can reduce pain scores and the demand for analgesic drugs 24 h postoperatively. Supra-inguinal FICB is accessed via a minimal risk approach to block the femoral nerve, lateral femoral cutaneous nerve, and obturator nerve, with rapid onset and definite analgesic effect, which procedure the anesthetization of the anterior, lateral, and medial areas of the thigh [16]. Wennberg [28] et al. reported that FICB effectively provided high-quality pain relief after THA.

It seems that both QLB and FICB cover similar parts of the fields. Cadaveric studies and clinical studies have shown that QLB leads to consistent blockade of branches of the lateral femoral cutaneous, ilioinguinal, iliohypogastric, and superior cluneal nerves and inconsistent anesthetization of the obturator, femoral nerve, and lumbar sympathetic trunk [19, 20]. FICB can produce a consistent sensory block of the femoral, obturator, and lateral femoral cutaneous nerves [16]. The combination of QLB and FICB, which is the high-and-low combination, can optimize nerve block effects from block range and degree. In our study, the patients in group QF had better pain relief, lower opioid requirements, and higher quality of recovery than patients in group Q. Additionally, the safety of T-QLB and FICB was higher than that of traditional techniques (such as lumbar plexus block). As the fascial plane block target is a fascial plane rather than a specific nerve (nerve root), this approach decreases the risk of nerve injury [29]. The injection site of the needle tip is more superficial, which reduces the risk of unrecognized blood vessel bleeding [30]. Furthermore, FICB is considered easy to learn and perform, and it can relieve patients’ pain when changing positions and ensure patients' comfort during the whole process.

Our results suggest that T-QLB combined with FICB can provide effective analgesia for up to 18 h. The prolongation of analgesia time seems to exceed the expectation of 0.375% ropivacaine in peripheral nerve blockade [31]. Multiple reasons account for these results. First, in our study, both QLB and FICB involved tissue (fascial) plane injections. The absorption rate of local anesthetics depends on local tissue perfusion [30]. Murouchi [32] et al. reported that the peak concentration of ropivacaine after QLB was lower than that of TAPB at a comparable time, and the duration of analgesia was significantly longer. Second, the procedure performed on individuals in group QF further reduced the sensitivity of nerves to surgical stimulation, prevented central and peripheral sensitization, and reduced or eliminated pain caused by nociceptive stimulation [33]. Last, patients' oral paracetamol 1 g regularly at 6 h intervals after operation also prolonged the time to the first opioid requirement.

The ability of the NRS to reflect the effect of pain control is limited due to the application of multimodal analgesia. In our study, we observed that there was no significant difference in NRS between the two groups at 18 h after surgery. Taking postoperative sufentanil consumption into account, we believe that the combination of QLB and FICB provides a more effective analgesic effect in control group Q, which mainly maintains a low pain score by increasing sufentanil consumption. Additionally, we applied the Qor-15 scale (scores from 0–10 for each term, where 0 = no existence, 10 = always existed. The higher the Qor-15 scale score, the better the recovery quality of patients) to evaluate recovery quality after surgery and anesthesia, including physiological comfort, physical independence, psychological support, emotion, and pain [23]. Our study shows a significant difference in the Qor-15 scale score and ROM between the two groups at 24 h and 48 h, consistent with a significant reduction in sufentanil consumption. Therefore, it further confirmed that the blockade combination contributes to relieving postoperative pain, reducing postoperative anxiety, improving patient satisfaction and comfort, and optimizing early postoperative recovery quality.

All blocks were performed before anesthesia induction. Hydroseparation of the target interfascial plane with saline is beneficial to the local anesthetic’s correct deposition and improves the block's success rate. Moreover, a professional investigator evaluated the analgesic effect 30 min after performing the nerve block to avoid potential block failure. In our study, three patients in group Q were excluded due to an ineffective block, which reduced the occurrence of selective bias.

It would be better for elderly patients with comorbidities to use an anesthetic with higher safety and longer half-life, such as ropivacaine [31]. In this study, 150 mg of ropivacaine was safe and effective for elderly patients. However, previous studies [14, 34] reported that complications such as hypotension and urinary retention were observed after performing QLB, which did not occur in our study. Future studies should focus on the minimal effective volume for proximal spreading and the dose–response relationship. Additionally, ropivacaine has the function of sensory-motor integration, and it can block the sensory nerve while retaining motor nerve function, which has significant advantages for the early recovery of postoperative patients [31].

We acknowledge that our study has some limitations. First, we did not use objective indicators to quantify the nerve block effect on muscle strength. However, the postoperative evaluation of motor function is difficult. The motor function may be affected by severe postoperative pain, iatrogenic nerve injury, and transient nerve palsy [35]. Therefore, it can be considered that the decrease in motor function postoperatively is not entirely caused by nerve block. Second, we evaluated the sensory block of the obturator, femoral, and lateral femoral cutaneous nerves in our study. However, we did not test the subcostal, ilioinguinal, and iliohypogastric nerve distributions as a part of the sensory assessment. Third, we did not investigate the time to first ambulation, length of hospital stay, patient satisfaction, or all-important outcome parameters for evaluating the efficacy of ERAS. Finally, we performed two different PNBs under general anesthesia for surgery usually performed under spinal anesthesia, which limited the applicability of the practice and the external generalizability of our results. Our findings are preliminary, and future research should investigate the effects of the combination of T-QLB and FICB under spinal anesthesia or local anesthetic infiltration techniques.

## Conclusion

In conclusion, ultrasound-guided T-QLB combined with FICB can be safely and effectively used in elderly patients undergoing total hip arthroplasty, achieve a multimodal analgesic effect with opioid-sparing, and improve the recovery quality.

## Data Availability

The datasets used and/or analyzed during the current study are available from the corresponding author on reasonable request.

## Abbreviations

THA: Total hip arthroplasty
PNB: Peripheral nerve block
PONV: Postoperative nausea and vomiting
T-QLB: Transmuscular quadratus lumborum block
FICB: Fascia iliaca compartment block
S-FICB: Supra-inguinal fascia iliaca compartment block
LA: Local anesthetic
ASA: American Society of Anesthesiologists
BMI: Body mass index
QLM: Quadratus lumborum muscle
PM: Psoas major
LMA: Laryngeal mask airway
PetCO2: Partial pressure of end-tidal carbon dioxide
BIS: Bispectral index
MAP: Mean arterial pressure
PACU: Post anesthesia recovery room
PCIA: Patient-controlled intravenous analgesia
NRS: Numerical rating scale
Qor-15: Quality of recovery-15
ROM: Range of motion
SD: Standard deviation
LMM: Linear mixed-effects model
